# DNA methylation perturbations may link altered development and aging in the lung

**DOI:** 10.18632/aging.202544

**Published:** 2021-01-19

**Authors:** Priyadarshini Kachroo, Jarrett D. Morrow, Carrie A. Vyhlidal, Roger Gaedigk, Edwin K. Silverman, Scott T. Weiss, Kelan G. Tantisira, Dawn L. DeMeo

**Affiliations:** 1 Channing Division of Network Medicine, Brigham and Women's Hospital, Boston, MA 02115, USA; 2 Children's Mercy Hospital and Clinics, Kansas City, MO 64108, USA; 3 Division of Pulmonary and Critical Care Medicine, Brigham and Women's Hospital, Boston, MA 02115, USA

**Keywords:** aging, DNA methylation, development, lung, transcription factors

## Abstract

Fetal perturbations in DNA methylation during lung development may reveal insights into the enduring impacts on adult lung health and disease during aging that have not been explored altogether before.

We studied the association between genome-wide DNA-methylation and post-conception age in fetal-lung (n=78, 42 exposed to in-utero-smoke (IUS)) tissue and chronological age in adult-lung tissue (n=160, 114 with Chronic Obstructive Pulmonary Disease) using multi-variate linear regression models with covariate adjustment and tested for effect modification by phenotypes. Overlapping age-associations were evaluated for functional and tissue-specific enrichment using the Genotype-Tissue-Expression (GTEx) project.

We identified 244 age-associated differentially methylated positions and 878 regions overlapping between fetal and adult-lung tissues. Hyper-methylated CpGs (96%) were enriched in transcription factor activity (FDR adjusted P=2x10^-33^) and implicated in developmental processes including embryonic organ morphogenesis, neurogenesis and growth delay. Hypo-methylated CpGs (2%) were enriched in oxido-reductase activity and VEGFA-VEGFR2 Signaling. Twenty-one age-by-sex and eleven age-by-pack-years interactions were statistically significant (FDR<0.05) in adult-lung tissue.

DNA methylation in transcription factors during development in fetal lung recapitulates in adult-lung tissue with aging. These findings reveal molecular mechanisms and pathways that may link disrupted development in early-life and age-associated lung diseases.

## INTRODUCTION

DNA methylation represents an epigenetic mark with a critical role in early life development and aging [1] that differs across tissues [2]. Previous studies in ageing research have emphasized adulthood, however increasing evidence has highlighted that age-associated cognitive decline as well as adverse later life health outcomes, for example, depend largely on epigenetic programming or adaptations during fetal development [3]. With the complex reprogramming during early-life stages, interplay of dynamic DNA methylation, chromatin marks and environmental effects during the life course make it further complicated to identify age-related versus cumulative life-course manifestations of the genome [4, 5]. Prenatal and lifetime exposures have a tremendous impact on both short- and long-term consequences mediated through epigenetic mechanisms and may contribute to a broad spectrum of immune responses, respiratory, cardiovascular and age-related diseases in later life [6, 7]. Evidence suggests impact of hypo- and hyper-methylation [8] including an altered epigenetic state on several age-associated neurological functions and human disease [9]. A recent meta-analysis identified several novel age-associated differentially methylated CpG sites in newborns and children reflective of processes critical to development [10]. Further efforts have identified the role of sex [11] and race-specific [12] differential methylation in blood along the aging process or associated pathologies. Fetal tissue DNA methylation changes associated with post-conception age and sex have been suggested to reflect brain development and are supportive of the idea that aging processes start early in life [13]. Previously, strong tissue-specific age associations with DNA methylation and gene expression have been identified in fetal and adult human livers [14], however, there is a paucity of previously reported tissue-specific studies in the literature and none for lung. Therefore, identifying more accurate biomarkers in lung tissue may merit further investigation to identify individuals at risk for age-associated lung function decline.

Recent studies have identified DNA methylation at selected CpGs to strongly predict chronological [15–21] age, constituting the epigenetic clock [21]. This may be especially useful to identify or differentiate individuals at the greatest risk for age-related health disparities and conditions. There is also a growing interest in understanding the role of epigenetic age acceleration or deceleration during the intrauterine period and adulthood. A significant association was observed between prenatal exposure to tobacco smoke and the risk of accelerated aging at birth [22], suggestive of developmental effects during childhood and the biomarker potential of age acceleration in adulthood [23]. However, limited agreement with low to moderate correlations have been observed between different epigenetic clocks estimating biological age owing to the various aspects captured by them including mortality risk, smoking, lifespan and time to death [21, 24].

To our knowledge, epigenetic links between fetal lung development and age-associated epigenetic marks in adult lung tissue (including DNA methylation age) have not been extensively explored. In this study, we performed a comparative analysis of age associated differential methylation and DNA methylation age in fetal and adult lung tissues and evaluated effect modification of age associations as a marker of development by sex and smoke exposure. Given that chronic lung diseases may be impacted by both altered lung development and accelerated aging processes, investigating age associated DNA methylation may inform new insights into lung diseases of aging, including chronic obstructive lung disease (COPD).

## RESULTS

### Age-associated differential methylation in fetal and adult lung tissues

**Figure 1:**
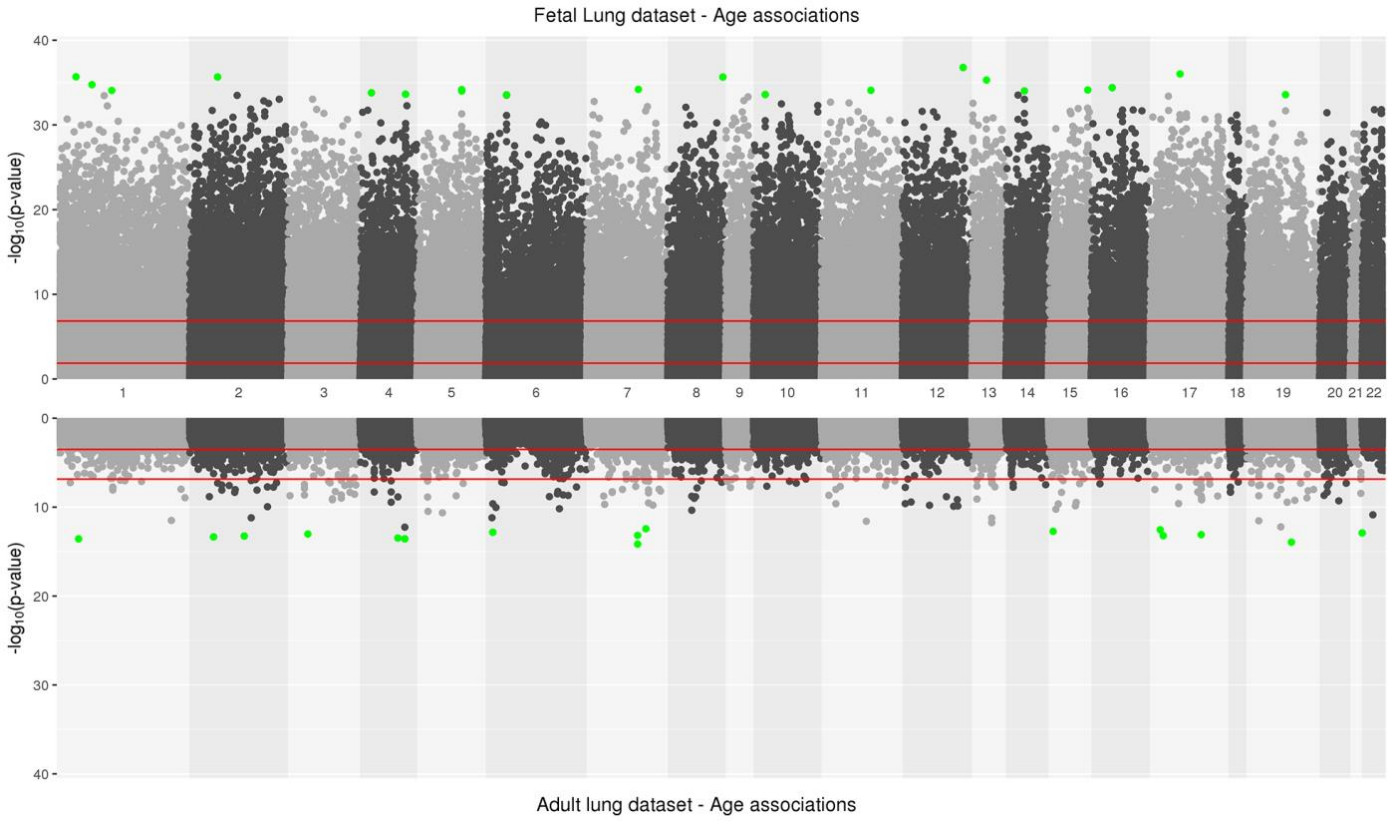
**Manhattan plot depicting significance on y-axis and distribution of CpGs across all chromosomes on x-axis for fetal lung dataset (top panel) and adult lung dataset associations with age (bottom panel).** Top 20 CpGs in both datasets have been highlighted in green. The two red lines represent the CpG sites significant at an FDR<0.05 and at a Bonferroni threshold (0.05/number of tests) in fetal and adult lung datasets.

The IUS-exposed and unexposed samples did not differ by mean gestational age of the percentage of males and females in the fetal lung dataset (Table 1). 94,834 CpGs (27.1%) were significantly associated with fetal age (FDR <0.05; Supplementary Table 1, Figure 1, Supplementary Figure 1) and 35,846 (10.3%) age-associated differentially methylated positions (aDMPs) remained at a Bonferroni significance threshold (P<1.43x10^-7^). Of the 94,834 CpGs, 40,836 sites were relatively hypo-methylated and 53,998 sites were relatively hyper-methylated with increasing age (Supplementary Figure 2A) and were strongly enriched in gene body and 3’UTRs (Hypergeometric P-Value<2.2x10^-16^, P-Value=5.4x10^-62^ respectively) regions. Of the top 20 significant age-associated DMPs, 11 were located in CpG shores and their top associated genes included *C17orf96* and *CASZ1* (Table 2A). Twelve CpGs were also significantly associated with age at an absolute difference in methylation of at least 1% and with most sites increasing in methylation with age; their top associated genes included *IGFBP1* and *MEGF11* (Supplementary Figure 2A). In our fetal lung aDMPs, we also found previously discussed differentially methylated age-associations identified in fetal brain tissue [13] including *SFRP1*, *NR4A2* and *SHANK2*.

**Table 1:** Overall clinical characteristics in fetal and adult lung tissue (ALT) datasets.

***Fetal lung tissue (n=78)***				
	**All subjects (n = 78)**	**IUS-Exposed (n = 42)**	**Unexposed (n = 36)**	**P-value**
Post-Conception Age (days), mean (SD)	88.7 (16)	86 (16.4)	91.9 (15.2)	0.1
Chronological Age (years), mean (SD)	-0.50 (0.04)	-0.50 (0.04)	-0.49 (0.04)	0.1
DNAmAge (years), mean (SD)	0.50 (0.30)	0.41 (0.28)	0.60 (0.30)	8.1x10^-3^
Age Acc. difference, mean (SD)	0.99 (0.27)	0.92 (0.25)	1.08 (0.27)	7.1x10^-3^
Age Acc. residual, mean (SD)	0.00 (0.20)	-0.04 (0.17)	0.05 (0.21)	0.03
Sex, n (%)				0.84
Female	28 (36)	16 (38)	12 (33)	
Male	50 (64)	26 (62)	24 (67)	
***Adult lung tissue (n=160)***				
	**All subjects (n = 160)**	**With COPD (n = 114)**	**Without COPD (n = 46)**	**P-value**
Age (years), mean (SD)	63.9 (7.4)	63.4 (6.7)	65.3 (8.9)	0.19
DNAmAge (years), mean (SD)	66.8 (6.6)	66.4 (6.2)	67.7 (7.4)	0.33
Age Acc. difference, mean (SD)	2.8 (5.1)	3 (4.4)	2.3 (6.7)	0.50
Age Acc. Residual, mean (SD)	0.00 (4.5)	0.01 (4)	-0.04 (5.5)	0.96
Sex, n (%)				0.31
Female	89 (56)	60 (53)	29 (63)	
Male	71 (44)	54 (47)	17 (37)	
Race, n (%)				0.78
African American	25 (16)	19 (17)	6 (13)	
White	131 (81.9)	92 (80.7)	39 (84.8)	
Others	4 (2.5)	3 (2.6)	1 (2.2)	
Time since quitting (months), mean (SD)	112 (113)	181 (139.9)	84.7 (87.2)	6.7x10^-5^
LAA-950, mean (SD)	0.3 (0.2)	0.3 (0.1)	0.0 (0.1)	<2.2x10^-16^
Pack-years	53.3 (27.8)	61.2 (26.4)	33.6 (20.5)	1.6x10^-10^

Significance of difference was evaluated using two-sample t-test for continuous variables and chi-squared test for categorical variables except race where fisher test was used due to smaller number of samples in individual categories. Missing data: Time since quitting data (months) was missing for 1 subject without COPD, fraction of lung voxels with low attenuation areas at less than -950 Hounsfield Units (LAA-950) was missing for 27 subjects without COPD and 28 COPD subjects.

Abbreviations: SD, standard deviation; n, number of subjects; DNAmAge, DNA methylation or epigenetic age; Acc., Acceleration.

**Table 2A:** Age-associated differential methylation in the fetal lung tissue datasets.

**CG site**	**UCSC Gene**	**CHR**	**MAPINFO**	**Island**	**Gene context**	**Coef**	**AveBeta**	**P-Value**	**Adjusted P**
cg14651082	*NA*	12	125,145,939	S_Shore		0.004	0.26	1.67E-37	5.82E-32
cg00964751	*C17orf96*	17	36,828,900	N_Shore	1stExon	0.005	0.32	9.66E-37	1.55E-31
cg01821043	*CASZ1*	1	10,766,044	S_Shore	5'UTR	0.005	0.60	2.09E-36	1.55E-31
cg03955927	*LOC100132215*	2	63,272,232	Island	Body	0.007	0.66	2.19E-36	1.55E-31
cg05457903	*PLEC1*	8	145,052,304	Island	TSS1500	0.004	0.28	2.23E-36	1.55E-31
cg03389701	*DLEU7*	13	51,418,221	S_Shore	TSS1500	0.004	0.47	5.03E-36	2.93E-31
cg25133130	*MAP3K6*	1	27,687,685	Island	Body	0.004	0.43	1.79E-35	8.93E-31
cg09018299	*TNRC6A*	16	24,748,339	OpenSea	Body	-0.006	0.72	4.01E-35	1.75E-30
cg24025567	*CUX1*	7	101,505,662	OpenSea	Body	0.003	0.47	6.38E-35	2.31E-30
cg11067714	*PURA*	5	139,492,230	N_Shore	TSS1500	0.006	0.34	6.74E-35	2.31E-30
cg14205663	*NR2F2*	15	96,872,827	N_Shore	TSS1500	0.005	0.29	7.48E-35	2.31E-30
cg25903143	*NA*	11	68,920,466	OpenSea		0.006	0.25	8.33E-35	2.31E-30
cg11654900	*NA*	1	51,444,685	S_Shore		0.009	0.26	8.58E-35	2.31E-30
cg13803234	*RAD51L1*	14	68,830,813	OpenSea	Body	0.005	0.50	9.65E-35	2.38E-30
cg00079023	*PURA*	5	139,492,535	N_Shore	TSS1500	0.003	0.18	1.02E-34	2.38E-30
cg10681992	*TBC1D14*	4	6,964,527	OpenSea	Body	0.004	0.62	1.62E-34	3.53E-30
cg05878390	*MAB21L2*	4	151,502,935	N_Shore	TSS200	0.007	0.23	2.36E-34	4.84E-30
cg19791727	*NA*	10	21,798,015	Island		0.008	0.55	2.57E-34	4.84E-30
cg22783327	*FXYD1*	19	35,633,258	N_Shore	Body	0.006	0.30	2.71E-34	4.84E-30
cg06519422	*GABBR1*	6	29,599,226	N_Shore	Body	0.004	0.46	2.82E-34	4.84E-30

Top 20 significant differentially methylated CpGs associated with age in the fetal lung data set. P-values were adjusted by the Benjamini-Hochberg method to correct for multiple testing.

Abbreviations: UCSC, The University of California Santa Cruz; Coef, regression coefficient - Adjusted mean difference (per day increase or decrease) in methylation with age. NA: un-annotated CpG site; CHR, chromosome; AveBeta, average beta; P, p-value.

In the adult lung tissue (ALT) dataset, COPD subjects differed from subjects without COPD by time since quitting cigarette smoking (in months), LAA-950 and pack-years but did not differ by age (Table 1). We identified 2,217 (0.7%) significant aDMPs (FDR <0.05, Supplementary Table 2, Figure 1). Of those, 2,181 were relatively hyper-methylated and 36 were relatively hypo-methylated with increasing age and a maximum absolute effect size of 0.7% per year (Supplementary Figure 2B). These aDMPs were strongly enriched for transcription start sites (Hypergeometric P-Value=2.1x10^-31^). The top 20 significant age-associated DMPs were mostly located in CpG islands; their top associated genes included *DLL3* and *PRDM2* (Table 2B). Two sites, cg16867657 (regression coefficient: 0.004, FDR adjusted P=3.8x10^-9^) and cg24724428 (regression coefficient: 0.006, FDR adjusted P=3.8x10^-9^) annotated to *ELOVL2* gene, a biomarker of age [25] and also the top gene based on effect size in the adult lung data (Supplementary Figure 2B).

**Table 2B:** Age-associated differential methylation in the adult lung tissue dataset.

**CG site**	**UCSC Gene**	**CHR**	**MAPINFO**	**Island**	**Gene context**	**Coef**	**AveBeta**	**P-Value**	**Adjusted P**
cg07640648	*DLL3*	19	39,993,697	Island	Body	0.001	0.04	1.15E-14	2.00E-09
cg26830108	*NA*	7	100,813,299	N_Shelf		0.002	0.07	7.02E-15	2.00E-09
cg02650266	*NA*	4	147,558,239	Island		0.003	0.13	2.73E-14	2.34E-09
cg05024939	*NA*	4	113,442,251	N_Shore		0.002	0.26	3.35E-14	2.34E-09
cg23813012	*PRDM2*	1	14,026,482	Island	TSS1500	0.002	0.10	2.70E-14	2.34E-09
cg05617798	*AURKB*	17	8,113,714	Island	5'UTR	0.001	0.07	6.07E-14	2.61E-09
cg21186299	*VGF*	7	100,808,810	Island	1stExon	0.001	0.03	6.73E-14	2.61E-09
cg23606718	*AMER3*	2	131,513,927	Island	5'UTR	0.003	0.24	5.61E-14	2.61E-09
cg27320127	*KCNK12*	2	47,798,396	Island	TSS1500	0.003	0.22	4.54E-14	2.61E-09
cg16477091	*PPM1E*	17	56,833,000	Island	TSS1500	0.002	0.15	8.06E-14	2.82E-09
cg10806820	*CELSR3*	3	48,699,090	Island	1stExon	0.003	0.22	9.47E-14	3.01E-09
cg12373771	*CECR6*	22	17,601,381	Island	1stExon	0.003	0.22	1.25E-13	3.65E-09
cg16867657	*ELOVL2*	6	11,044,877	Island	TSS1500	0.004	0.74	1.49E-13	3.76E-09
cg24724428	*ELOVL2*	6	11,044,888	Island	TSS1500	0.006	0.26	1.50E-13	3.76E-09
cg01763090	*OTUD7A*	15	31,775,406	N_Shore	3'UTR	0.002	0.12	1.89E-13	4.40E-09
cg15906794	*YBX2*	17	7,197,963	Island	TSS200	0.004	0.17	2.84E-13	6.22E-09
cg02383785	*NA*	7	127,808,848	Island		0.003	0.22	3.68E-13	7.57E-09
cg05991454	*NA*	4	147,558,435	Island		0.003	0.12	5.58E-13	1.08E-08
cg07544187	*CILP2*	19	19,651,235	Island	Body	0.004	0.15	6.05E-13	1.11E-08
cg03036557	*GPC5*	13	92,050,720	N_Shore	TSS1500	0.002	0.07	1.79E-12	3.13E-08

Top 20 significant and differentially methylated CpGs associated with age in the adult lung tissue dataset. P-values were adjusted by the Benjamini-Hochberg method to correct for multiple testing.

Abbreviations: UCSC, The University of California Santa Cruz; Coef, regression coefficient - Adjusted mean difference (per year increase or decrease) in methylation with age. NA: un-annotated CpG site; CHR, chromosome; AveBeta, average beta; P, p-value.

From the aDMPs identified in the adult lung dataset, 244 CpGs (11% of the differentially methylated loci) that mapped to 144 unique genes (13.7% of the unique differentially methylated genes) overlapped with the fetal lung age-EWAS (Supplementary Table 3). The corresponding findings from the analysis stratified by IUS-exposure and COPD status in fetal and adult lung datasets for these 244 CpGs have been included in Supplementary Table 3. In the fetal lung dataset, the effect estimates for 238 CpGs from the non-IUS-exposed and all 244 CpGs from the IUS-exposed samples only analysis were in the same direction to the effect estimates of the 244 overlapping aDMPs from the overall fetal lung analysis. In the ALT dataset, 243 CpGs from the non-COPD subjects only and all 244 CpGs from the COPD case-only analysis were in the same direction of effect to the effect estimates of the 244 overlapping aDMPs from the overall ALT analysis (Supplementary Table 3).

The top 20 annotated CpGs were associated with nine transcription factors: *EVX1*, *HIF1A*, *ALX3*, *SATB2*, *PITX2*, *NKX2-4*, *ZIC1*, *ZIC2, BSX* and are presented in Table 2C. Of those 244 CpGs, 215 had effect estimates in the same direction (210 hyper- and five hypo-methylated) including the CpG site cg16867657 mapped to the promoter of *ELOVL2* gene. The methylation patterns for cg16867657 were progressively hyper-methylated with age for both the fetal and adult lung datasets (Supplementary Figure 3A, 3B).

**Table 2C:** Age-associated differential methylation in the fetal and adult lung tissue datasets.

					**Fetal lung**			**Adult lung**			
**CG site**	**CHR**	**MAPINFO**	**UCSC Gene**	**Context**	**Avebeta**	**Coef**	**Adjusted P**	**AveBeta**	**Coef**	**Adjusted P**	**Direction**
cg22296612	6	15,457,699	*JARID2*	Body	0.92	0.002	3.60E-15	0.99	-0.0001	0.016	+-
cg17110767	1	243,637,966	*SDCCAG8*	Body	0.67	0.003	4.24E-13	0.97	0.001	0.014	++
cg20801476	7	27,281,465	*EVX1*	TSS1500	0.06	0.001	5.27E-13	0.24	0.001	0.048	++
cg20580088	14	62,161,583	*HIF1A*	TSS1500	0.13	0.001	2.28E-12	0.28	0.002	0.012	++
cg17611674	10	8,094,431	*FLJ45983*	Body	0.10	0.001	2.34E-12	0.38	0.002	0.020	++
cg24388061	8	141,249,678	*TRAPPC9*	Body	0.73	0.002	7.82E-12	0.94	0.001	0.008	++
cg21595709	19	15,344,186	*EPHX3*	TSS1500	0.05	0.001	2.90E-11	0.21	0.001	0.039	++
cg00484358	1	110,610,995	*ALX3*	Body	0.12	0.001	1.07E-10	0.38	0.001	0.039	++
cg27583307	2	200,320,750	*SATB2*	Body	0.03	0.000	2.35E-10	0.11	0.001	0.049	++
cg03470772	4	85,503,328	*CDS1*	TSS1500	0.03	0.000	4.48E-10	0.26	0.002	0.028	++
cg20992114	4	111,542,825	*PITX2*	Body	0.08	0.001	7.98E-10	0.28	0.001	0.002	++
cg07247419	20	21,376,484	*NKX2-4*	3'UTR	0.02	0.000	1.45E-09	0.24	0.002	0.007	++
cg22197050	2	63,276,183	*LOC100132215*	TSS1500	0.03	0.000	5.83E-09	0.27	0.002	0.000	++
cg06306198	3	147,128,998	*ZIC1*	Body	0.05	0.001	1.73E-08	0.23	0.001	0.042	++
cg11814235	18	28,621,490	*DSC3*	Body	0.02	0.000	1.97E-08	0.12	0.001	0.031	++
cg14614094	9	133,567,903	*EXOSC2*	TSS1500	0.36	0.001	3.80E-08	0.69	0.002	0.015	++
cg15110296	12	12,509,705	*LOH12CR1*	TSS1500	0.68	-0.002	5.41E-08	0.26	-0.003	0.042	--
cg18431640	13	100,637,191	*ZIC2*	Body	0.03	0.000	1.45E-07	0.19	0.001	0.008	++
cg24719321	11	122,850,490	*BSX*	Body	0.02	0.000	1.53E-07	0.18	0.002	0.002	++
cg08376141	6	32,116,591	*PRRT1*	3'UTR	0.11	0.001	2.11E-07	0.53	0.003	0.003	++

Top 20 annotated and differentially methylated age-associated DMPs overlapping between fetal lung and the adult lung tissue dataset. P-values were adjusted by the Benjamini-Hochberg method and are sorted based on the discovery fetal lung dataset.

Abbreviations: UCSC, The University of California Santa Cruz; Coef, regression coefficient - Adjusted mean difference (per day increase or decrease for fetal lung dataset and per year increase or decrease for adult lung tissue dataset) in methylation with age. NA: un-annotated CpG site; CHR, chromosome; AveBeta, average beta; P, p-value.

Considering aging may have a genetic component, we had further investigated the presence of methylation quantitative trait loci (mQTLs) among the overlapping 244 aDMPs using the previously published methylation lung tissue QTL results [26]. Sixty-three (25.8%) and 17 (7%) of the 244 aDMPs in both fetal and adult lung datasets overlapped with the 1,787 significant cis mQTLs (located within 1 Mb of a specific CpG site) and 133 significant trans mQTLs (located beyond 1 Mb of a specific CpG site or on different chromosomes) at an FDR < 0.05 (Supplementary Table 4) which provide evidence for genetically influenced aDMPs. Interestingly, among the overlap with cis-mQTLs, the top gene based on significance was *FADS2*; its associated polymorphisms modulate fatty acid metabolism [27] and influence asthma risk [28]. However, the smaller overlap with mQTLs suggests that majority of our age associations (67.2%) were not genetically driven.

The regional analysis in fetal and adult lung datasets resulted in 14,427 and 270 significant regions (FDR<0.05) respectively with an overlap of three regions by exact chromosomal coordinates and 878 regions (Supplementary Table 5) by overlapping genes. Two of those regions with exact overlap of chromosomal coordinates mostly mapped to small nucleolar RNAs (snoRNAs/*SNORA*) and small cajal body-specific RNAs (scaRNAs/*SCARNA*) family genes, while the third region could not be mapped to any genes.

### Effect modification of age-associated methylation by smoke exposure and sex

In the fetal lung data, the interaction between gestational age and exposure to cotinine (continuous variable) resulted in 11,810 differentially methylated CpGs (P<0.05); none were robust to adjustment at an FDR of 5% (FDR<0.05) or less stringent threshold of 10% (FDR<0.10) but many passed at a nominal P-value of 0.05 (Supplementary Table 6). This is consistent with the modest findings from our prior IUS “main effects” analysis [29]. Interaction between gestational age and sex revealed three CpGs. relatively hyper-methylated with age in males compared to females (FDR<0.10, Supplementary Table 7).

In the adult lung data, the interaction of chronological age with time since smoking cessation resulted in one differentially methylated CpG site mapped to the *DSCC1* gene (cg21745419; regression coefficient =3.3x10^-6^; FDR adjusted P=3.9x10^-3^). The interaction between age and pack-years resulted in 10 differentially methylated CpGs significant at an FDR threshold of 0.05 (Supplementary Table 8). One CpG site: cg10682155 mapped to the *SIM2* gene and overlapped with the nominal age by exposure associations in fetal lung (Supplementary Table 7). Interaction of age with sex resulted in 47 significant differentially methylated sites (FDR<0.10, Supplementary Table 8); 21 of those CpG sites were also significant at an FDR threshold of 0.05. Interestingly, cg24724428 mapped to the *ELOVL2* gene promoter and also demonstrated age-by-sex associations (regression coefficient=0.007; FDR adjusted P=0.06). This CpG was 10 bases upstream of the age-associated *ELOVL2* CpG site cg16867657. These analyses demonstrated potential methylation effect modification with age by both sex and smoke exposure in fetal and ALT datasets.

### CpGs associated with age are enriched in transcription factor pathways

**Figure 2:**
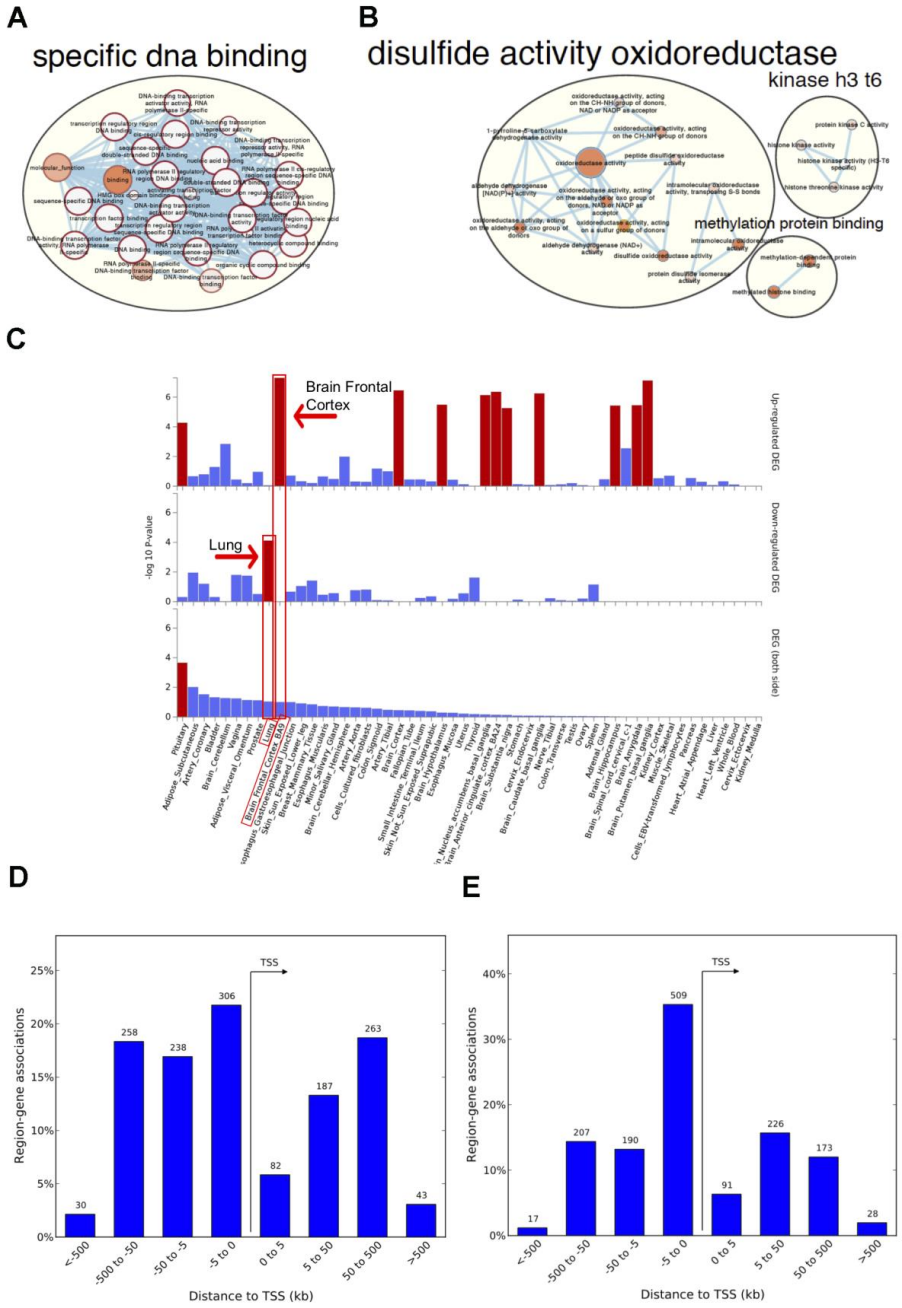
**Network visualization, functional enrichment and region-gene associations in both fetal and adult lung tissue datasets.** Network clusters of the molecular function gene ontology terms and annotated pathways including reactome and wikiPathways were created using the gene symbols mapped to the significant and age-associated differentially methylated positions (DMPs) overlapping between fetal and adult lung datasets. (**A**) Hyper-methylated CpGs were mainly enriched in transcription factor DNA binding whereas (**B**) hypo-methylated CpGs were enriched in oxidoreductase activity. Size corresponds to the overlap of genes between the enriched terms and color corresponds to significance. The analysis for hypo-methylated CpGs was limited by few numbers of genes represented by few CpGs (**C**) Functional enrichment of age-associated hyper-methylated DMPs in both fetal and adult lung tissue datasets amongst differentially expressed genes in 54 GTEx tissues (x-axis) and –log10(*P*-value) on the y-axis. Tissues with significant gene enrichment (FDR<0.05) are highlighted by red bars and tissues with the highest enrichment amongst the downregulated and upregulated genes are highlighted by arrows (**D**) Region-Gene associations using chromosomal coordinates for the differentially methylated regions in fetal lung dataset (**E**) Adult lung tissue dataset. TSS: transcription start site.

Genes annotated to hyper-methylated aDMPs in both fetal and adult lung tissue datasets had the most significant enrichment for RNA polymerase II-specific DNA-binding transcription factor activity (FDR adjusted P=2.0x10^-33^; 52 genes; Supplementary Table 9, Figure 2A). Genes annotated to hypo-methylated aDMPs were mainly enriched (FDR<0.05, Figure 2B) for Cell Cycle (*PRKCA, NCAPD3*), oxidoreductase activity (*ALDH4A1, PDIA6*) and VEGFA-VEGFR2 signaling pathway (*PRKCA, PDIA6*), however this analysis may have been limited due to the small number of genes represented by few overlapping hypo-methylated aDMPs between both datasets. Most enriched biological processes in the overlapping aDMPs between fetal and ALT included terms related to development including embryonic organ morphogenesis, neurogenesis and growth delay. Two enriched wiki-pathways were identified: Neural Crest Differentiation annotated with five genes (*ZIC1, HAND1, OLIG3, ZIC5, PAX3*) and Mesodermal Commitment Pathway annotated with six genes (*PITX2, ZIC2, HAND1, PAX6, ZIC5, SOX21*).

The 125 genes with age associations in the same direction in both fetal and ALT datasets were further assessed for gene-based enrichment analysis and functional relevance in gene-sets using tissue-specific expression in 54 tissue types from the Genotype-Tissue Expression project (GTEx) v8 [30]. Among the tissue-specific gene-sets, the strongest enrichment for hyper-methylated CpGs was observed among the down-regulated genes in the lung; up-regulated genes were noted for brain regions but not for lung (Figure 2C, Supplementary Table 10). Among the curated gene-sets and transcription factor targets obtained from MsigDB, the strongest enrichment was for poly-comb regulated genes in human embryonic stem cells, predominantly H3K27me3 and transcription factor binding sites respectively (Supplementary Figure 4A, 4B). The most enriched transcription factor target gene-set included *PAX6, HOXC4, FOXG1, SALL3, TBR1, DLX1, ZIC4, ZIC1, SHOX2, ATOH1, PITX2* and *OLIG3* (Supplementary Figure 4C) with hyper-methylated CpGs in our findings. There was no tissue-specific or gene set enrichment observed for genes annotated to hypo-methylated CpGs, likely due to small numbers. Gene associations for the significant differentially methylated regions also had the most significant enrichment for RNA polymerase II transcription factor activity/sequence-specific DNA binding (Supplementary Table 11) with a significant percentage of those within 5kb of transcription start site for both fetal and ALT datasets (Figure 2D, 2E).

### Measures of epigenetic age acceleration in fetal and adult lung tissues

We did not identify a large overlap between the 353 Horvath clock markers and our differentially methylated age EWAS associations. Of the 353 CpG clock markers, 91 markers existed within the fetal lung aDMPs and 17 within the adult lung aDMPs and two CpGs (cg12946225: *HMG20B*; cg23517605: *TUBB2B*) were common to all three comparisons. Our EWAS quality control processing pipeline filtered out some of those 353 clock markers, however the smaller overlap may also suggest a different subset of age associations captured between the models.

Among the fetal lung samples, chronological age was significantly positively correlated with the DNAmAge as expected (cor=0.76, P=3.6x10^-16^, Supplementary Figure 5A). Overall, the fetal lung sample ages appeared epigenetically accelerated with higher age estimates for DNA methylation age compared to chronological age (Table 1). However, IUS-exposure was associated with lower epigenetic age differences when compared to epigenetic age of unexposed samples after adjusting for available covariates, suggesting relative developmental delay related to IUS exposure (Estimate: -0.10; 95% CI: -0.19, -0.01; P=0.03; Table 3). In the multivariate regression model including age and other covariates, we did not detect statistical differences by sex although they were in the same direction of effect as in the ALT dataset (Table 3).

**Table 3:** Associations between epigenetic age acceleration and lung phenotypes; IUS-exposure and sex in fetal lung dataset and COPD status, sex, time since quitting (in months), pack-years and fraction of lung voxels with low attenuation areas at less than -950 Hounsfield Units (LAA-950) in the adult lung tissue dataset.

***Epigenetic clock metrics in fetal and adult lung tissues***		
	**Age Acceleration**	
**Multivariate model predictors**		
**Fetal Lung**	**Estimate (95% CI)**	**P-value**
IUS-exposure	-0.10 (-0.19, -0.01)	0.03
Sex	0.03 (-0.07, 0.12)	0.57
**Adult Lung**	**Estimate (95% CI)**	**P-value**
COPD	2.20 (-1.04, 5.44)	0.18
Sex	1.98 (0.13, 3.83)	0.036
months-quit	-0.01 (-0.02, 0.004)	0.21
pack-years	-0.01 (-0.05, 0.02)	0.46
LAA-950	-1.94 (-9.30, 5.43)	0.60

Multivariate models were residualized for age and were additionally adjusted for potential confounders in both datasets. In fetal lung dataset, unexposed samples were used as reference compared to IUS-exposed samples; covariates included post-conception/gestational age (continuous) and sex (male or female; female as reference). In adult lung tissue dataset, subjects without COPD were used as reference compared to COPD subjects; covariates included chronological age, sex (male or female; female as reference), race (white, African-Americans and others), pack-years (smoke exposure, continuous), LAA-950 (marker for airway emphysema, continuous), months-quit, (time since quitting in months, continuous) and technical covariate sample plate. DNAmAge as output from Horvath clock (in years) was used for both datasets. Age acceleration was calculated as the difference between DNAmAge and chronological age. Significant P-Values are highlighted as bold.

Abbreviations: CI, confidence interval.

Missing data: Time sine quitting data (months) was missing for 1 subject without COPD, fraction of lung voxels with low attenuation areas at less than -950 Hounsfield Units (LAA-950) was missing for 27 subjects without COPD and 28 COPD subjects.

In the adult lung dataset, we observed significant positive correlation between chronological age and the DNAmAge (cor=0.74, P<2.2x10^-16^; Supplementary Figure 5B). The COPD cases showed a trend for accelerated aging compared to subjects without COPD (Estimate: 2.20; 95% CI: -1.04, 5.44; P=0.18, Table 3); however males demonstrated significant epigenetic age acceleration compared to females that stayed robust after adjustment with covariates in the multivariate model (Estimate: 1.98; 95% CI: 0.13, 3.83; P=0.036, Table 3).

Investigating smoking cessation behaviors, there was a suggestive trend for association between age acceleration and recent smoke cessation only within females compared to males in the multivariate model. The trend was not significant in males, however the effect estimates were in the same direction (Supplementary Table 12).

## DISCUSSION

Aging and age-associated epigenetic drift [31] in prenatal and postnatal life drive progressive and widespread methylation changes that can be captured by epigenetic clocks [16], however there is no clear gold standard [21]. Telomere shortening had long been considered a hallmark of biological aging, however recently weak correlation was found between telomere length and age-associated health outcomes [32]. With refinement in aging algorithms, ongoing efforts for precision health initiatives aim to identify better epigenetic biomarkers with predictive potential for obstructive lung disease, as these may facilitate primary interventions [33]. Our study importantly contributes to identifying age-associated patterns of differential methylation overlapping between early-life (fetal) and adulthood (adult lung) implicated in crucial developmental processes and demonstrates their effect modification by sex and smoke-exposure. This has never been examined before in lung tissue studies.

Rapid and considerable changes in methylation occur in early fetal life; therefore our finding of thousands of age-associated DMPs in fetal lung is not surprising. One of the most interesting and encouraging global findings was enrichment of hyper-methylated CpGs for transcription factor activity and down-regulated genes in the lung from GTEx gene-sets. Increased tissue-specific transcriptional variability has been associated with age, with DNA methylation playing an integral role in activating or silencing a gene mediated by transcription factor binding [34]. Evidence also suggests age-associated hypermethylation in whole blood to occur at chromatin promoter domains typical of transcriptional repression in nearby neural genes [35] and those that are involved in DNA binding and transcriptional regulation [36]. Aberrant hypermethylation and silencing of genes required for maintenance of a differentiated state has been observed in murine lung development and lung cancer [37]. A 2012 healthy aging study [38] pointed out that the age-associated DMPs and DMRs that are often activated or deregulated early in life hinted towards developmental defects that may not necessarily represent healthy aging in later life and cause a wide range of detrimental health outcomes. As an example, one of the several transcription factors that we mentioned was *ZIC1*. The tissue-specific transcription factor *ZIC1* and its family functions by binding to enhancer regions in the developing brain and was identified as having a crucial regulatory role in neuronal differentiation and age-related Alzheimer's disease [39]. When looking specifically at region-overlap, the majority of those genes included snoRNAs/scaRNAs. These non-coding RNAs have gained substantial interest lately due to their clinical relevance in various pathologies including lung cancer, host antiviral responses and their regulatory role in RNA modifications, splicing and telomerase activity [40]. Moreover, it was reassuring that our overlapping age-associated DMRs between both fetal and adult lung tissue datasets were also enriched in DNA-binding transcription factor activity with common gene-sets, which may represent regions of regulatory potential as well as targets of epigenetic therapy. Of course, functional transcription factor binding can only be confirmed via experimental validation. However these results do suggest that methylation changes associated with aging and environmentally mediated exposures throughout life may impact biological processes and pathways crucial for growth, development and immune system regulation during the life course. Considering directionality of effect, most of our adult EWAS findings between both fetal and adult lung datasets were hyper-methylated with age which is in line with the previous findings [38] pointing towards the methylation hotspots that are consistently altered with age-dependent or age-related phenotypes.

Age associated epigenetic variation may additionally reveal genes further disrupted in age associated lung diseases such as COPD. In our fetal lung aDMPs, we found previously discussed differentially methylated age-associations identified in fetal brain tissue [13] including the Wnt antagonist *SFRP1* known to be differentially methylated in adults with asthma [41] and previously associated with emphysema [42], nuclear receptor gene *NR4A2* crucial for neurogenesis and *SHANK2* implicated in neurodevelopmental disorders such as autism and severe asthma [13, 43]. *ELOVL2* is a consistently identified biomarker for aging [12] further supported by our finding of association of the age-related *ELOVL2* gene and the rapid hypermethylation of its promoter cg16867657 [25, 44]. Interestingly, we also identified an age-by-sex association for the *ELOVL2* gene, at another nearby CpG site: cg24724428; both these sites were significantly correlated with age in the EWAS Atlas and in the majority of tissues [44].

Age associated enrichment of hypo-methylated sites characterized by methylation protein binding, oxido-reductase and aldehyde dehydrogenase activity while limited with few CpGs in our study could point to oxidative stress that can cause susceptibility to inflammatory disorders of the airways such as asthma and COPD. Variable methylation and expression changes have previously linked aging, COPD [45] and idiopathic pulmonary fibrosis (IPF) [46]. Of note, genes in VEGFA-VEGFR2 signaling mapped to hypo-methylated CpGs. The vascular endothelial growth factor-A and its family is crucial for endothelial cell survival and angiogenesis and its components are known biomarkers for asthma-COPD overlap syndrome [47]. One of the blood-based studies in lung tissue associated smoking with site-specific hypo-methylation [48]. The age-associated sites modified by smoke exposure and smoking cessation may further attenuate the risk of age-related lung diseases such as COPD and IPF or may point to reversed methylation signatures by smoking cessation over time for fetal origins of lung disease and susceptibility to other developmental or neurological disorders triggered in early life. There is also evidence that imbalance in the redox potential mediates inflammation, airflow limitation and airway remodeling in asthma and COPD [49]. Cigarette smoking is known to cause oxidative stress, disrupting the vascular endothelial growth factor (VEGF)/VEGF receptor (VEGFR) signaling and may promote emphysema [49]. These findings provide mechanistic insights into lung tissue-specific signatures of hypo- and hyper-methylation with age, pointing to a sensitive transcriptional regulation mediated by DNA methylation modifications during in-utero periods of developmental plasticity and later life.

We further examined epigenetic age acceleration or deceleration during the critical prenatal exposures and in later life. Our study highlighted epigenetic clock deceleration in IUS-exposed compared to unexposed samples. Prenatal exposure to hypoxic conditions has been associated with epigenetic age deceleration [50], alterations in immune and inflammatory responses, developmental delay and cognitive decline in later life [51]. In ALT, we observed age acceleration in males compared to females. This is in line with previously reported age acceleration in males over females corresponding with higher all-cause male mortality [12, 23, 51]. We also observed a suggestive trend in ALT for more recent smoking driving epigenetic age acceleration suggesting that smoking cessation could improve respiratory and lung health through modulation of lung aging pathways, a finding of potential public health relevance further supporting efforts to curb inhalational exposures starting early in life.

These findings suggest two plausible aging mechanisms [22]: (i) disruption to the aging processes may begin before birth, and (ii) certain prenatal exposures might increase/decrease the disease risks through perturbations in aging pathways as a defense or adaptation mechanism. In accordance with this hypothesis, decreased epigenetic age was associated with longevity in supercentenarians and their offspring [52] but also cerebroplacental ratio, a marker for fetal adaption to hypoxic conditions and adverse pregnancy outcome [50].

We acknowledge that our study also has limitations including the sample sizes of the lung datasets, paucity of longitudinal lung tissue data, limited phenotype data including race, geography, socioeconomic status in the fetal lung dataset and potential residual confounding by cellular heterogeneity associated with the lung samples even after surrogate variable adjustment. Evidence of high genomic inflation, despite adjusting for confounders as we have in this study, has also been observed and discussed frequently in other age EWAS studies and in principle could be used as a marker of development and biological age [11, 53, 54]. Moreover, limited association in fetal and adult lung tissue with the epigenetic clock metrics could be due to lack of a true association or the consideration that a lung specific clock would perform better, given the richness of the age associated EWAS findings. However, investigating age-associated methylation signatures using a robust analytical framework, in unrelated fetal and adult lung tissue samples with a broader age range including critical stages of development as well as adulthood, strengthens our study. Of note, it is unlikely that one particular omic-type or few genetic variants could lead to complex diseases of lung. Therefore, integrative omics analyses with larger sample sizes would mostly support future sensitivity or sex-stratified analyses and contribute to the greatest extent in identifying causal genes and pathways.

In summary, our findings point to age-related differential methylation that may serve as a starting point for similar future studies to advance our understanding of age-associated epigenetic programming and aid development of more sensitive lung based epigenetic age estimator. From a public health standpoint, considering overlapping developmental and aging pathways as potential targets for future interventions early in life may have relevance to curb the incidence of complex chronic diseases with the aging of the global population. Our study combines aging and epigenetic signatures and may constitute a system to promote lung health and longevity by evaluating age-modifying interventions across the life course.

## MATERIALS AND METHODS

### Study samples

Fetal lung DNA samples (n=78, 42 exposed to in utero maternal cigarette smoke (IUS), 36 non-IUS-exposed) were isolated from discarded tissue from 57-122 days of gestation as previously described [29, 55]. We performed genome-wide methylation profiling on these samples using the Illumina HumanMethylation450 BeadChip. Fetal sex was confirmed using unique Y chromosome microarray probes and verified using X and Y chromosome methylation. IUS exposure was assessed by measuring placental cotinine concentrations [56]. Exposure was treated as a dichotomous variable (1/0 with 1=exposed), with levels of cotinine < 7.5 ng/g considered as unexposed and levels of cotinine > 7.5 ng/g as exposed, though we also assessed the continuous cotinine variable. Approval was obtained from the Partners Human Research Committee Institutional Review Board in Boston, MA.

We used our previously published [57] adult lung tissue (ALT) dataset, from the Adult Lung Tissue study (n=160; 114 COPD cases, 46 subjects without COPD) to investigate age-associated differential methylation in adulthood. Genetic data was available for a subset of these samples. This led to removal of eight samples from the fetal lung dataset and 11 samples from the ALT dataset for whom we could not calculate ancestry principal components due to unavailability of the data. This applied to all the epigenome-wide association (EWAS) models.

### Epigenome-wide age association and statistical analyses

The proportion of DNA methylation at any CpG site reported as the Illumina beta (β)-value is defined as the ratio between methylated signal intensity and total probe signal intensity of methylated and un-methylated signal. A β-value ranges between 0 and 1 where 0 is considered an un-methylated CpG site and a value approaching 1 is considered a completely methylated CpG site. For biological interpretation purposes, we used beta-scale for all models [58]. Normalization and preprocessing were performed in Bioconductor [59] package Minfi [60]. We evaluated site based differential methylation (outcome) by age in both fetal lung and adult lung datasets with linear regression using limma [61] with correction for multiple testing using Benjamini-Hochberg method. We accounted for the following available covariates from the fetal lung data: IUS-exposure to cotinine (0: unexposed as reference, 1: exposed subjects) and sex (male/female with female as reference) and sample plate and sentrix position as technical covariates. Correcting for sentrix position accounts for the positional effects of the samples on the array [62]. For adult lung data, we included the following covariates: COPD disease status (cases/subjects without COPD as reference), sex (male/female as reference), race (white as reference versus black and others), pack-years (continuous) and sample plate as a technical covariate. The models evaluating age-associated differential methylation were further tested with interaction terms to assess effect modification by smoke exposure and sex. Ancestry composition was estimated from genetic data using the TRACE program (fasT and Robust Ancestry Coordinate Estimation) as implemented in the LASER package: (http://csg.sph.umich.edu/chaolong/LASER/) [63]. For the fetal and ALT datasets, we adjusted for genetic confounding using the first three (12.3%) and two (8.3%) principal components (PCs) respectively that explained most variability in the data. PCs did not reveal any confounding by row and column for the ALT dataset so sentrix position was not included in the model. Additionally, we accounted for unknown technical confounders in the fetal lung dataset such as observed due to cell-type heterogeneity using supervised version of surrogate variable analysis (s-sva) [64].

Differentially methylated positions (DMPs) were categorized as hyper- or hypo-methylated with a unit increase in chronological age (per day for fetal lung and per year for adult lung dataset). We further evaluated age-related differential methylation for effect modification and included interaction terms of age with IUS-exposure (categorical variable), cotinine levels (continuous variable) and sex in the fetal lung dataset and pack-years, sex, COPD, time since quitting/smoking cessation (in months) and fraction of lung voxels with low attenuation areas at less than -950 Hounsfield Units (LAA-950) to assess emphysema in the adult lung dataset. For main effects, we used a False Discovery Rate (FDR) of less than or equal to 0.05 and for interaction results, we relaxed thresholds for exploration and used an FDR of 10% (FDR<0.10). CpG sites were mapped to genes within which they were located using Human Genome build: GRCh37/hg19 and Bioconductor annotation package: IlluminaHuman Methylation450kanno.ilmn12.hg19 [65] and their gene context annotations were provided. If the CpG sites were not mapped to a gene, only their CpG island annotations were provided. We identified the overlapping age-associated differentially methylated positions/CpGs (aDMPs) between fetal and ALT datasets at probe level and at an FDR < 0.05. As a sensitivity analysis, we additionally evaluated the age associations in fetal and ALT datasets after stratifying by IUS-exposure and COPD status respectively. Considering aging may have a genetic component, we further investigated the presence of methylation quantitative trait loci (mQTLs) among these aDMPs using the previously published methylation lung tissue QTL results [26].

Region-based analyses was performed using the kernel smoothing method DMRCate [66]. Differentially methylated regions (DMRs) were calculated from log transformed CpG values (M-values) and defined as regions with at least two significant sites separated by a maximal distance of 1000 base pairs at an FDR of less than or equal to 0.05.

### Enrichment of disease associations and pathway analysis

Functional gene-set enrichment [30] and visualization of tissue-specific gene clusters was performed for significant aDMPs overlapping between fetal and ALT datasets using FUMA [30]. Unique gene symbols were mapped to Entrez IDs using Bioconductor package org.Hs.eg.db [67]. Network representation of clusters for the enriched gene ontology terms and pathways was performed using AutoAnnotate [68] and EnrichmentMap [69] apps in Cytoscape 3.7.1 [70]. Biological processes and pathways connected by edges were retained in the final network. Over-representation and pathway analysis of genes annotated to overlapping sites and regions was performed using gProfiler2 [71] package in R and Genomic Regions Enrichment of Annotations Tool (GREAT) [72] version 4.0.4 (http://great.stanford.edu/public/html/) respectively.

### DNAmAge and measures of epigenetic age acceleration

The Horvath clock [16] captures DNA methylation changes in chronological age using multiple tissue types and implies the epigenetic patterns from birth to the current age and included lung and fetal tissues during algorithm development. Therefore, we chose the Horvath clock method to determine the epigenetic age (DNAmAge) (http://labs.genetics.ucla.edu/horvath/dnamage/) [16] for the complete set of fetal (n=78) and adult lung samples (n=160) and additionally investigated differential methylation of the 353 CpG markers that form the aging clock. We computed two methylation-based age acceleration measures: age acceleration difference (epigenetic age – chronological age) and residual (linear regressing DNA methylation age on chronological age). Using age acceleration difference, we tested associations with IUS-exposure in fetal lung, and lung phenotypes in ALT. For all regression models, chronological age was included as a covariate [16]. To keep the units comparable to the chronological age in adult lung, post-conception age (in days) in fetal lung dataset was converted to a negative chronological age (in years) implying before birth: Chronological Age (in years) = Post Conception Age – (9*30)/365. In ALT dataset, correlation of epigenetic age acceleration with time since smoke cessation/quitting (in months) and LAA-950 was performed using Pearson correlation test. A positive difference between calculated epigenetic age and chronological age was considered accelerated aging and a negative difference decelerated aging.

### Ethics approval and consent to participate

Institutional Review Board approval was obtained at the three centers (Brigham and Women’s Hospital (Boston, MA), St. Elizabeth’s Hospital (Boston, MA), and Temple University Hospital (Philadelphia, PA)) for adult lung tissue dataset. Human fetal lung tissues were obtained from two NICHD-supported tissue retrieval programs at the University of Washington Center for Birth Defects Research (Seattle, WA) and the University of Maryland Brain and Tissue Bank for Developmental Disorders (Baltimore, MD). Approval was obtained from the Partners Human Research Committee Institutional Review Board at Brigham and Women’s Hospital in Boston, MA who declared the use of these tissues non-human subject research (2010-P-002399). Written informed consent was obtained from all participants. Our research was performed in accordance with the principles of the Helsinki Declaration.

## Supplementary Material

Supplementary Figures

Supplementary Table 1

Supplementary Table 2

Supplementary Table 3

Supplementary Table 4

Supplementary Table 5

Supplementary Table 6

Supplementary Table 7

Supplementary Table 8

Supplementary Table 9

Supplementary Table 10

Supplementary Table 11

Supplementary Table 12
