# Supplementary material for: DNA methylation perturbations may link altered development and aging in the lung
Source: Aging (Albany NY). 2021 Jan 19;13(2):1742–64. doi: 10.18632/aging.202544 (PMC7880367; doi:10.18632/aging.202544)
Supplement: Supplementary Figures [file aging-13-202544-s001.pdf]

SUPPLEMENTARY FIGURES

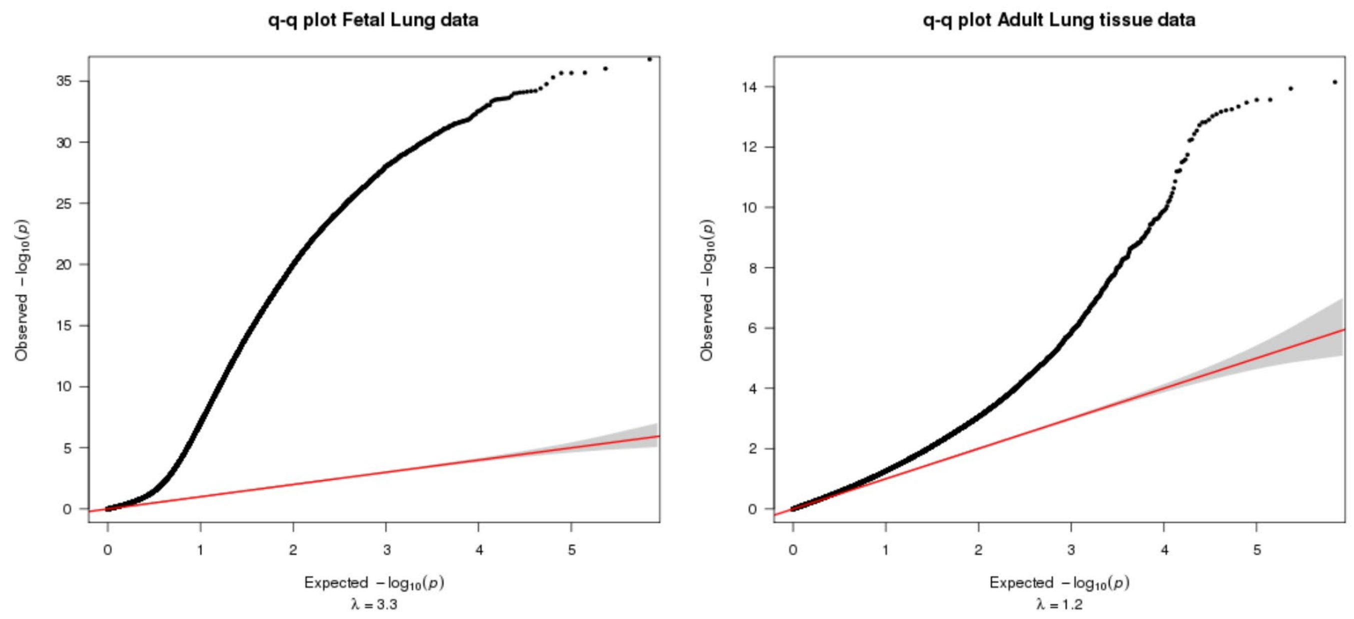

Supplementary Figure 1. Q-Q plot for fetal (left panel) and adult lung tissue (right panel) dataset.

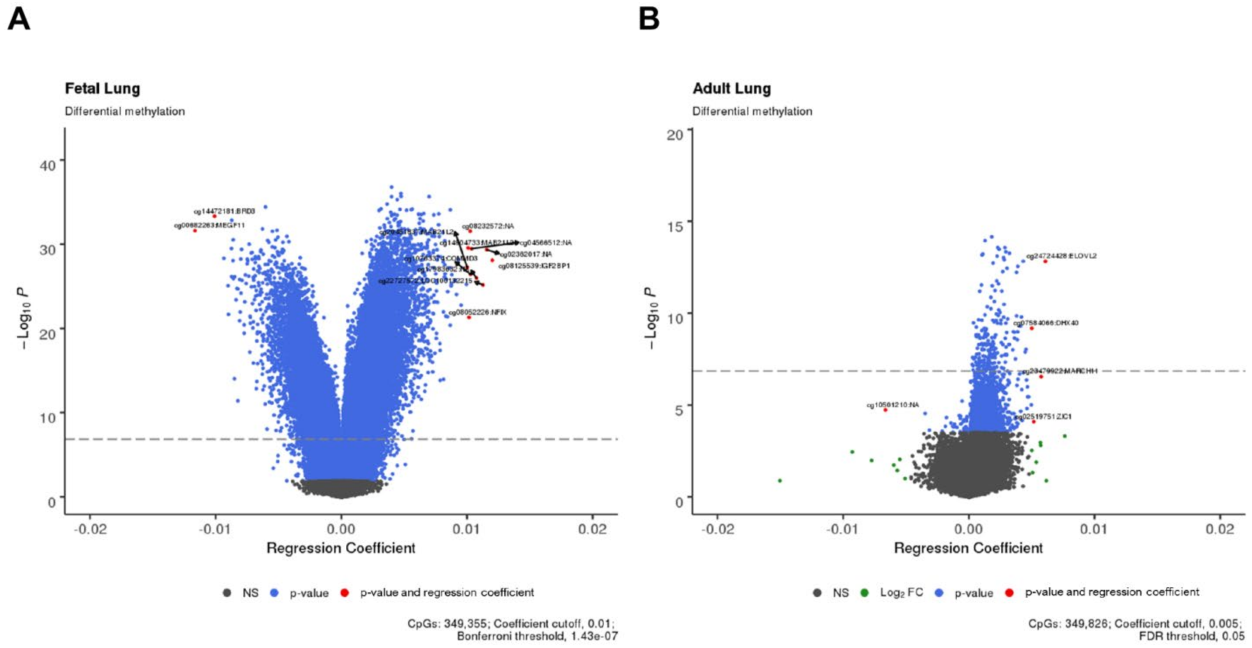

Supplementary Figure 2. Volcano plot depicting the effect size on x-axis and – log10P-Value on the y-axis. The blue colored dots are the CpG sites at an FDR adjusted P-Value of 0.05. (**A**) Fetal lung dataset. Gray line represents the cut-off for the age-associated DMPs at the Bonferroni threshold of 1.43×10^{-7}. Highlighted CpG sites as red dots and associated genes are at an absolute regression coefficient threshold (effect size) of 0.01. (**B**) Adult lung tissue (ALT) dataset. Gray line represents the cut-off for the age-associated DMPs at the Bonferroni threshold of 1.43×10^{-7}. Highlighted CpG sites as red dots and associated genes are at an absolute regression coefficient (effect size) threshold of 0.005. CpG sites colored green are those that passed the effect size threshold, however were not significant. Legend

abbreviations: Black dots as NS, not significant; Blue dots as p-value: age-associated DMPs at FDR<0.05; Red dots as p-value and regression coefficient, represented by difference in methylation with per day increase in age in fetal lung and per year increase in ALT dataset.

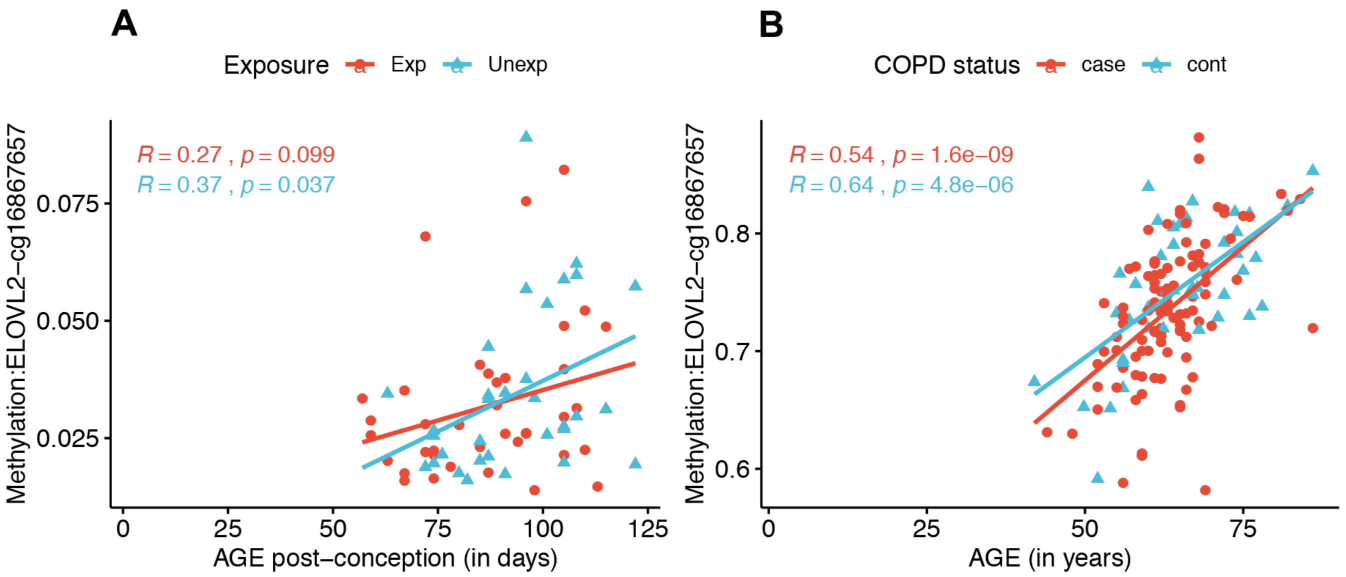

Supplementary Figure 3. Age-associated differential methylation of cg16867657 located within *ELOVL2* gene promoter for fetal lung (**A**) and adult lung tissue (**B**) datasets.

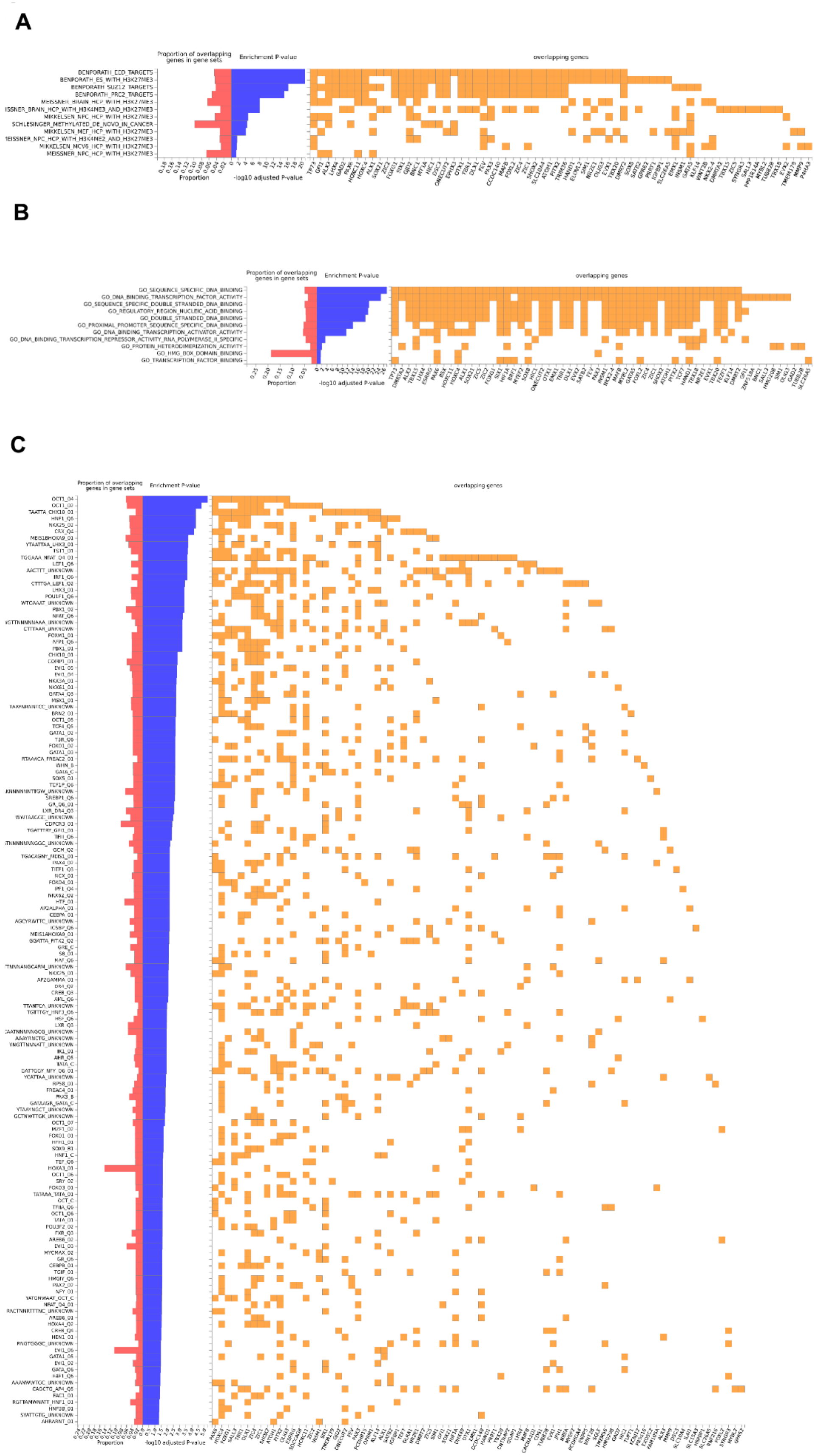

Supplementary Figure 4. Gene-set enrichment analysis for the genes annotated to the hyper-methylated age-associated DMPs overlapping between fetal and adult lung tissue datasets using curated gene-sets and transcription factor targets

obtained from MsigDB. (**A**) Enrichment based on Chemical and Genetic perturbation gene-sets (MsigDB). As an example: BENPORATH_EED_TARGETS signify genes identified by ChIP as targets of the Polycomb protein embryonic ectoderm development (EED) [GeneID=8726] in human embryonic stem cells. This protein mediates repression of gene activity through histone deacetylation and may act as a regulator of integrin function. (**B**) Enrichment based on GO molecular function gene-sets (MsigDB) (**C**) Enrichment based on gene-sets from TF targets (MsigDB). As an example, OCT1_04 signifies genes having at least one occurrence of the transcription factor-binding site (v7.4 TRANSFAC) in the regions spanning up to 4 kilo-bases around their (TSS) transcription starting sites.

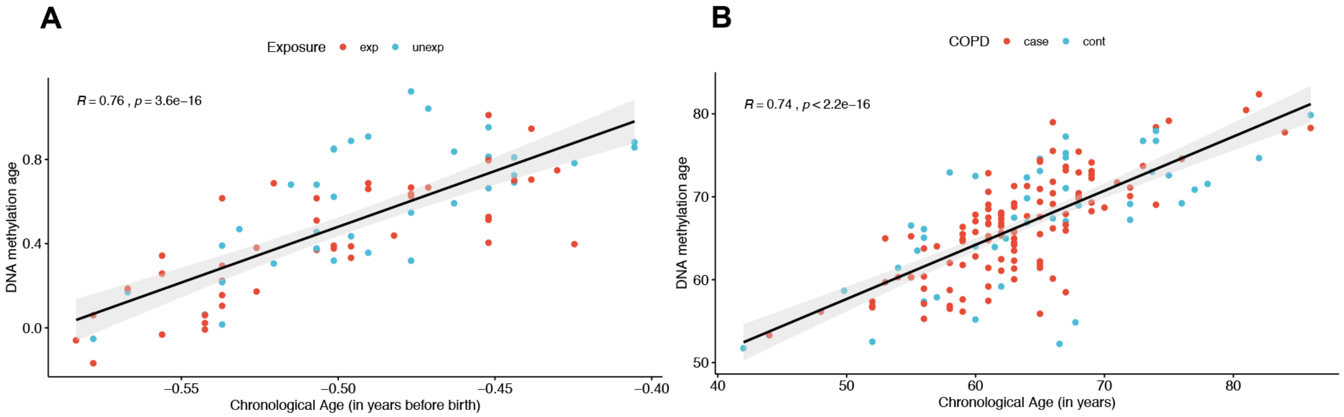

Supplementary Figure 5. Scatter plot of correlation between chronological age on x-axis and estimated DNAmAge (in years) on y-axis. Red: IUS-exposed fetal lung samples and COPD cases, Blue: Unexposed samples and controls respectively in fetal lung (**A**) and adult lung tissue (**B**) datasets. For fetal lung datasets, chronological age in days post conception was also converted to years (refer the paper for details), therefore x-axis represents before birth chronological age in negative.
