# Supplementary material for: DNA methylation perturbations may link altered development and aging in the lung
Source: Aging (Albany NY). 2021 Jan 19;13(2):1742–64. doi: 10.18632/aging.202544 (PMC7880367; doi:10.18632/aging.202544)
Supplement: Supplementary Table 12 [file aging-13-202544-s013.pdf]

SUPPLEMENTARY TABLE

Supplementary Table 12. Association between epigenetic age acceleration and adult lung tissue dataset phenotype: recent smoke cessation stratified by sex: in females and males.

	Age Acceleration	
**Multivariate model predictors**		
**Females**	**Estimate (95% CI)**	**P-value**
months-quit	-0.01 (-0.02, 0.001)	**0.068**
**Males**	**Estimate (95% CI)**	**P-value**
months-quit	-0.01 (-0.02, 0.01)	0.3

Note: Multivariate models were residualized for chronological age and were additionally adjusted for race, pack-years, LAA-950 and technical covariate plate.
Abbreviations: CI, confidence interval; percentage of low attenuation areas at less than -950 Hounsfield Units (LAA-950).
Missing data: Time sine quitting data (months) was missing for 1 subject without COPD.
